# Cu–Gallate MOF–Chitosan Hybrid Membrane for Low-Power, Non-Invasive Acetone Sensing: Toward Early-Stage Diabetes Detection

**DOI:** 10.1021/acsomega.5c09912

**Published:** 2025-11-24

**Authors:** Lamia A. Siddig, Yaser E. Greish, Ashraf Ali, Khadega A. Al-Maqdi, Abdul Hakeem Deshmukh, Naser N. Qamhieh, Saleh T. Mahmoud

**Affiliations:** † Department of Physics, 11239 United Arab Emirates University, Al-Ain 15551, United Arab Emirates; ‡ Department of Chemistry, United Arab Emirates University, Al-Ain 15551, United Arab Emirates; § Department of Physics, 105955 Khalifa University of Science and Technology, Abu Dhabi P.O. Box 127788, United Arab Emirates

## Abstract

The precise and noninvasive detection of volatile organic compounds (VOCs) is of growing importance in medical diagnostics, health monitoring, and environmental assessment. Among these VOCs, acetone in human breath serves as a key biomarker for the early detection of diabetes. In this work, we report the development of a high-performance composite membrane sensor based on a copper gallate metal–organic framework (Cu-gallate MOF) integrated with chitosan (CS) and doped with ionic liquid glycerol (IL). The incorporation of chitosan significantly improved membrane conductivity and structural stability, while hydrogen-bonding interactions enhanced acetone selectivity. As a result, the Cu-gallate MOF/CS/IL sensor achieved a remarkable detection limit of 0.25 ppm at an operating temperature of 80 °C, with rapid response (27 s) and recovery (10 s) times. The sensor further demonstrated excellent selectivity, long-term durability, and reproducible performance under practical testing conditions. These findings highlight the potential of MOF-based mixed-matrix membranes as a cost-effective and energy-efficient platform for real-time breath analysis, providing a promising pathway toward early diagnosis and monitoring of diabetes.

## Graphical abstract

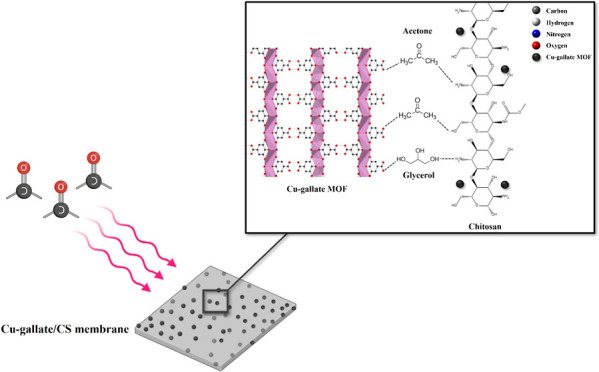

## 1 Introduction

The detection of acetone gas has emerged as a crucial tool in the noninvasive diagnosis and monitoring of diabetes. Acetone is one of the primary volatile organic compounds (VOCs) present in the breath of diabetic patients, and its concentration is closely linked to blood glucose levels. Elevated breath acetone levels can serve as an early biomarker for diabetic ketoacidosis. Compared with traditional blood-based methods, breath analysis offers a painless, rapid, and patient-friendly alternative, promoting better disease management. Thus, the development of highly sensitive and selective sensors for detecting acetone gas is important for advancing diabetes care.^1–3^

Various materials have been explored for gas sensing applications, including carbon-based materials,^4–6^ polymers,^7,8^ TMDs (transition metal dichalcogenides),^9^ MXene,^10,11^ MOFs (metal–organic frameworks),^12,13^ and MOSs (metal oxide semiconductors).^14–17^ However, each material has its limitations. Carbon-based sensors exhibit poor selectivity, reproducibility, and slow response times,^5^ while polymer-based sensors suffer from poor stability and selectivity.^18^ Although TMDs offer a high specific surface area and unique properties upon bandgap tuning, they generally exhibit sluggish sensor response times, poor sensing performance, and instability due to surface oxygen/moisture interactions.^9^ In contrast, MOS-based gas sensors, which feature diverse nanostructures, are widely used due to their advantageous characteristics including superior physicochemical properties, high response, small size, affordability, and ease of use. However, most of MOS sensors still face challenges such as high-power consumption, the need for elevated operating temperatures (200–600 °C), and interference from environmental humidity.^19–24^ Nevertheless, recent advances have demonstrated that several MOS-based sensors can achieve high response and low detection limits (below 100 ppb) toward breath acetone at temperatures below 100 °C.^25–28^

Metal–organic frameworks (MOFs) are favorable contenders for gas sensor technology owing to excellent chemical and thermal stability, high porosity, and large surface area.^29–33^ These crystalline organic–inorganic hybrid materials are composed of organic ligands surrounded by metal ions or metal oxide clusters, creating highly porous structures with numerous reaction sites. MOFs offer superior electrical conductivity and adsorption enthalpy for various gases, making them highly suitable for gas sensing applications. Their integration into gas sensor devices creates a synergistic effect, enhancing performance.^34–38^

Acetone is a volatile and flammable liquid with a low boiling point (∼56.5 °C), which is widely used in laboratory and industrial settings.^1,39^ Exposure of higher acetone vapor concentration (>173 ppm) for longer time can lead to nausea and eye/skin irritation, and in severe cases, it can potentially damage the central nervous and respiratory systems at higher acetone concentrations exceeding 300–500 ppm.^40–45^ Acetone detection in human breath is particularly important, as it acts as a biomarker for early diagnosis of diabetes.^46^ In individuals with diabetes, breath acetone levels can surpass 1.8 ppm, whereas the typical range for the healthy individuals falls between 0.2 and 1.8 ppm.^47^ However, human breath consists of a complex mixture of volatile gases and high humidity levels, making selective acetone detection crucial for accurate diabetes diagnosis. Therefore, developing efficient, rapid, and reliable methods for subppm acetone sensing is of utmost importance.^48,49^

Recently, various nanostructured materials have been employed for acetone detection, e.g., SnO_2_/Pd–NiO (SPN) nanowires, Co_3_O_4_/SnO_2_ nanofibers, etc.^50,51^ The SPN nanowire sensor demonstrated a 14.88% response to 500 ppm of acetone gas with a response time of 11 s at 450 °C, whereas the Co_3_O_4_/SnO_2_ nanofibers showed a higher response (∼216%) to 100 ppm at 350 °C within 0.62 s. Alternatively, MOFs have been predominantly used as precursors to synthesize functional MOS nanostructures for acetone sensing.^52,53^ At first, the MOF is converted into metal oxide through thermal annealing processes, affecting its structure and surface properties. Liu et al.^52^ transformed MIL-100­(Fe) MOFs into the Fe_2_O_3_ via annealing at various temperatures (330–550 °C) to evaluate their performance in detecting acetone gas. The resulting α-Fe_2_O_3_-based sensor demonstrated effective sensing capabilities within a higher temperature range of 100–300 °C, with optimal performance, i.e., detection limit of 1 ppm, response time of 4 s, and recovery time of 37 s for 100 ppm acetone observed at 250 °C. Also, the ZnO/ZnFe_2_O_4_ was fabricated from the Fe^III^-modified Zn-based MOFs via direct pyrolysis for acetone detection.^53^ For 5 ppm acetone concentration, the optimum gas sensing response of 9.4% was observed at 250 °C. However, for real-world applications such as breath analysis and environmental monitoring, acetone is supposed to be detected at very low temperatures to make them more practical and energy efficient in nature.

In this work, we developed a stable Cu–gallate MOF-based acetone gas sensor. To optimize the MOF performance, we intentionally integrated CS/IL as an organic matrix to enhance conductivity and improve gas-sensing properties. Additionally, its biocompatibility and NH_2_ functional groups facilitate hydrogen bonding with acetone gas.^13^ The sensor’s ability to selectively detect acetone is linked to its hydrogen-bonding structure and the inherently polar nature of acetone.^54^ We believe that the combination of Cu–gallate MOF and CS/IL provides a low-cost, simple, low-operating temperature, and efficient platform for breath analysis, contributing to the advancement of noninvasive diabetes detection. While direct breath analysis typically requires room-temperature sensing, the current sensor is more suited for preconditioned sampling systems that allow mild heating to enhance performance.

## 2 Materials and Methods

All chemicals were used as received without any additional purification. Cu­(NO_3_)_2_·3H_2_O, 3,4,5-trihydroxybenzoic acid (gallic acid), dimethylformamide (DMF), ethanol, and acetic acid were provided by Sigma-Aldrich Co. Glycerol, serving as an ionic liquid (IL), was supplied by Quarek Corp. Chitosan (CS) with 50,000–190,000 Da (≥75%) molecular weight was supplied by Polysciences, Warrington.

X-ray diffraction (XRD) patterns were recorded on a Rigaku MiniFlex benchtop diffractometer (Japan) using a Cu Kα source with λ = 1.542 Å at 40 kV. The samples were scanned at a rate of 2° per minute across a 2θ range from 3° to 50°. Thermogravimetric analysis (TGA) was conducted using a Shimadzu TGA-50 instrument, with samples placed in an aluminum pan. The analysis was performed under a nitrogen atmosphere at a flow rate of 50 mL/min, employing a 10 °C/min heating rate. Scanning electron microscopy (SEM) images were acquired via a Quattro ESEM system (USA), operating under high vacuum conditions at 30 kV with a magnification of 5000×. The SEM system was further equipped with an energy-dispersive X-ray (EDX) detector for elemental analysis. A Thermo Nicolet Nexus 470 spectrophotometer was used to acquire Fourier-transform infrared (FT-IR) spectra, where the KBr pellet technique was used to prepare samples for FTIR analysis. FTIR spectral 2 cm^–1^ resolutions were measured in the 4000–500 cm^–1^ range.

### 2.1 Synthesis of Cu–Gallate

The synthesis of Cu–gallate MOF was carried out according to a previously reported method.^55^ First, 0.376 g of gallic acid (2 mmol) was added to 5 mL of a solvent mixture consisting of DMF, ethanol, and H_2_O. Then, 0.232 g of copper­(II) nitrate hemipentahydrate (1 mmol) was dissolved in 5 mL of the same solvent mixture. The two solutions were then combined while stirring continuously before changing the solution pH to ∼6.5 via dropwise addition of 5 M KOH. Afterward, the reaction mixture was heated in a sealed autoclave for 12 h at 120 °C. The resulting sample was centrifuged three times using methanol over the course of 2 days, followed by three washes with dichloromethane (DCM) over 1 day to facilitate solvent exchange. MOF was activated by heating at 130 °C for 24 h under vacuum.

### 2.2 Cu–Gallate/CS/IL-Based Membrane Fabrication

To prepare the Cu–gallate-based membrane, a 20 mL solution was first prepared in a 100 mL beaker, consisting of 5 vol % ionic liquid (IL) and 3% acetic acid. Subsequently, 0.6 g of Cu–gallate MOF (3 wt %) and 0.4 g of chitosan were added and stirred continuously for 24 h at 25 °C. After mixing, the resulting solution was poured into a Petri dish to dry the samples in an oven at 70 °C for 18 h. The final membrane obtained was flexible, uniform, and black in color having 0.20 mm thickness (Figure 1(A) and (B)).

**Figure 1:**
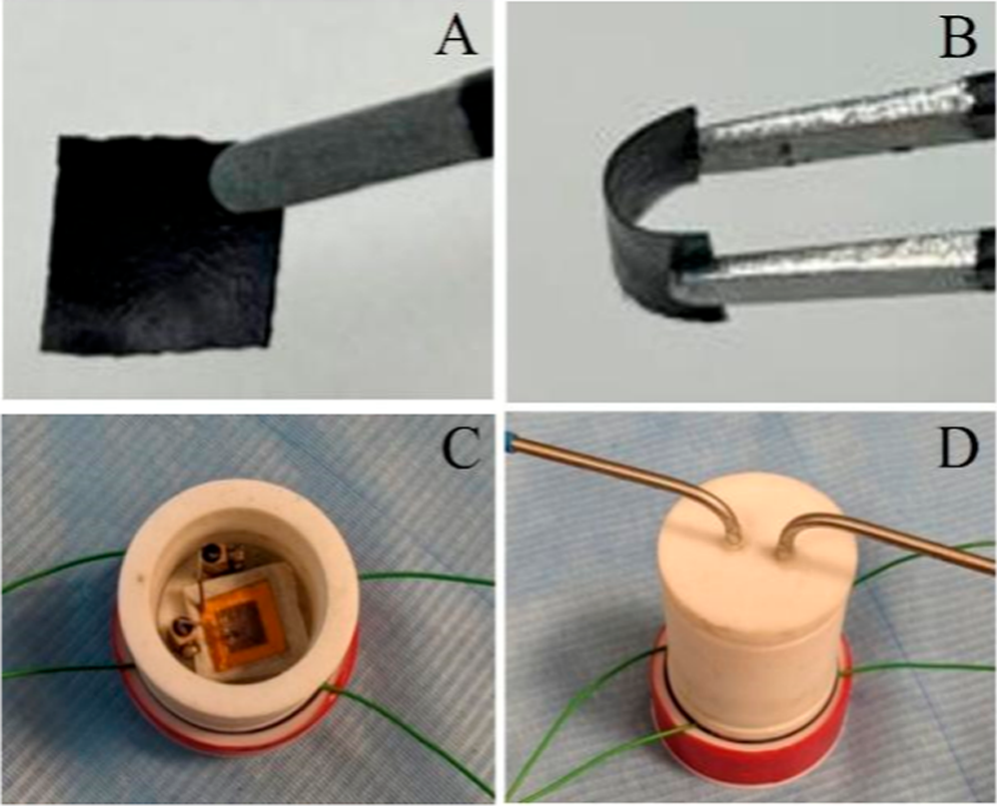
(A) 1 × 1 cm^2^ Cu–gallate MOF/CS/IL membrane, (B) membrane flexibility, (C) sensor device configuration including electrical connections and test chamber, and (D) a photograph of a custom-designed Teflon-based gas sensing chamber.

### 2.3 Device Fabrication and Gas Sensing

A prototype device was constructed using a sandwich approach where 1 × 1 cm^2^ (Figure 1A,B) of the fabricated membrane was placed between the bottom electrode (copper metal plate with 0.15 μm thickness) and top electrode (stainless-steel mesh with a size of 250 × 250 μm^2^), as demonstrated in our previous research.^13,37^ The stainless-steel mesh was used for the top contact due to anticorrosive properties. The temperature-resistant kapton tape was wrapped around three layers and then positioned inside a Teflon chamber attached to mass flow controllers via the electrical probes (Figure 1(C) and (D)). The MFCs diluted the test gas with synthetic air to the programmed concentration and delivered the mixture into the gas test. The target gas concentration was varied from 0.25 to 100 ppm in 200 sccm of the carrier gas. The setup was additionally connected to a temperature controller for the measurement of gas sensing at different temperatures, i.e., 25, 40, 60, and 80 °C.

For safety, gas sensing measurements using the custom-designed system were performed in the fume hood. The setup was kept dry and the tube was securely sealed to prevent gas leaks. During testing, the procedure involved alternating cycles of exposure of the sample to the target gas and synthetic air, ensuring that any remaining test gas molecules were completely cleared after each cycle.

## 3 Results and Discussion

PXRD data were obtained for the Cu–gallate MOF and the Cu–gallate/chitosan/IL membrane to confirm their purities and phase structure. Figure 2 displays the XRD patterns for both materials: the blue pattern represents the MOF, while the red pattern corresponds to the membrane. The PXRD data for both materials indicate high crystallinity, with sharp peaks observed in the 2θ range of 5–50°. Both materials exhibit the same pattern, consistent with the previously reported Cu–gallate MOF structure,^55,56^ with no additional impurity peaks. The diffraction peaks for both the PXRD patterns appeared at 10.1, 13.2, 20.1, 28.0, 31.1, and 42.8° which are the typical signatures of Cu–gallate structure, which aligns well with reported data.^55,56^ These results confirm the successful synthesis of a highly crystalline and pure MOF.

**Figure 2:**
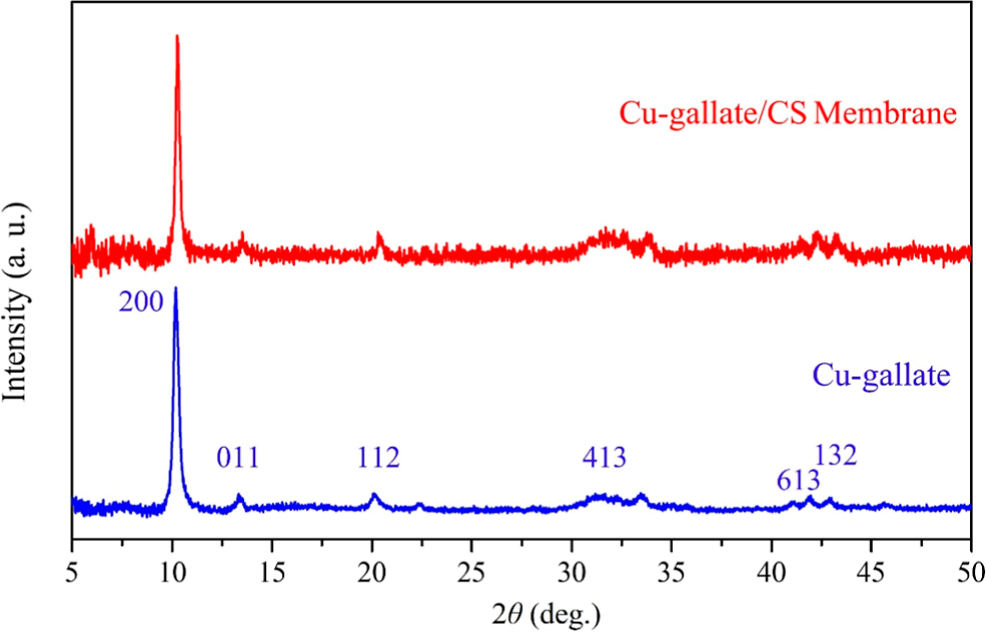
PXRD patterns of the synthesized Cu–gallate and Cu–gallate/CS/IL composite membrane.

The SEM images were acquired to investigate the morphologies of the prepared materials. In Figure 3A and 3B, the SEM images of the Cu–gallate/CS/IL membrane demonstrate successful integration and uniform MOF dispersion within the CS/IL matrix. EDX analysis (Figure S1 and Table S1) confirms the presence of copper, carbon, and oxygen in the MOF. Meanwhile, the membrane shows the same elements, with an increase in carbon and oxygen contents due to the addition of the CS compound. However, the percentage of Cu in the membrane decreased due to the low content of Cu–gallate MOF within the composite matrix.

**Figure 3:**
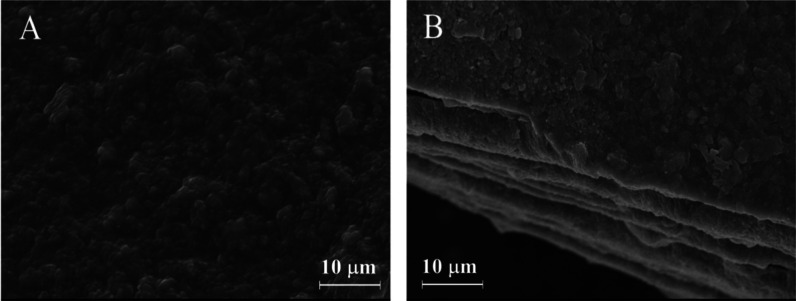
SEM image of (A) Cu–gallate/CS/IL membrane and (B) cross-section of the Cu–gallate/CS/IL membrane.

The thermal stabilities of chitosan, Cu–gallate, and Cu–gallate/CS/IL membrane were examined using TGA and DTG in the 25–600 °C temperature range (Figures 4 and S2). The Cu–gallate MOF (blue spectrum) displayed two steps of weight loss. The first step occurred at 70 °C with a 6% weight loss, due to the evaporation of water (either coordinated or occupied within MOF pores). The major weight loss was observed at 266 °C, accounting for 41% of the material, and could be assigned to the degradation of the organic linker (gallic acid). These observations are consistent with findings from previous studies.^55^ Complete decomposition resulted in a plateau of 53 wt %, corresponding to the formation of copper oxide.

**Figure 4:**
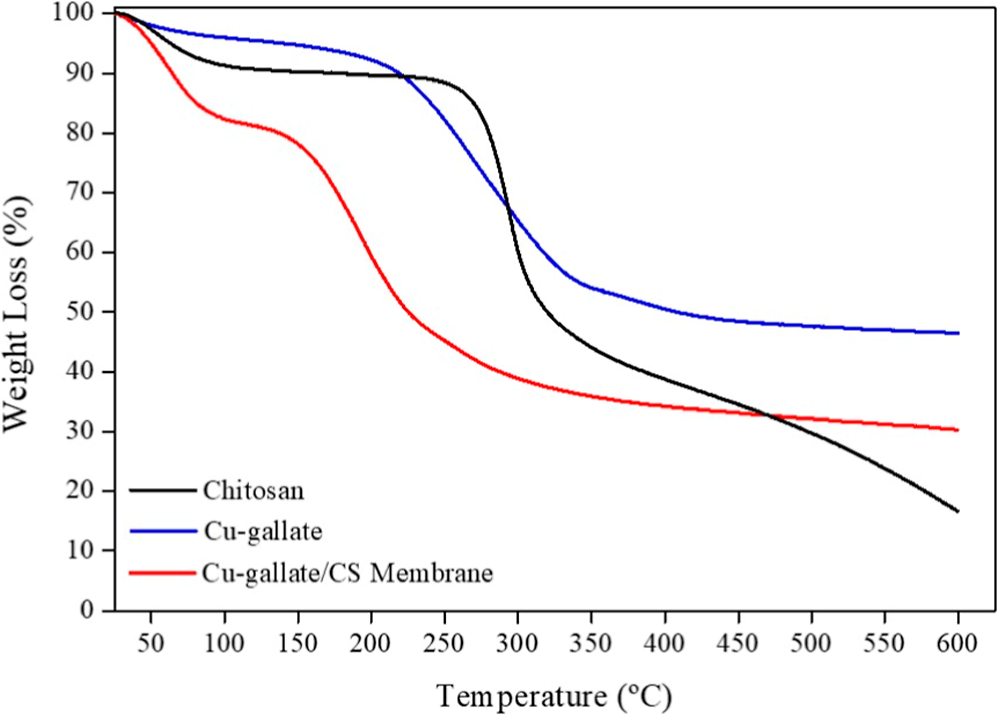
Thermogravimetric analysis of chitosan, Cu–gallate MOF, and Cu–gallate/CS/IL membrane with a heating rate of 10 °C/min^–1^.

In contrast, the TGA and DTG curves of the chitosan matrix (black spectrum) showed two weight loss events (at 58 and 293 °C), corresponding to the evaporation of water (both physically or chemically absorbed) and the chitosan degradation. The Cu–gallate/CS/IL membrane displayed thermal events at 61 and 193 °C with weight losses of 18% and 42%, respectively. The membrane exhibited an ultimate loss of 60% of its weight by 600 °C, resulting in the complete decomposition of the prepared membrane.

The binding modes and chemical interactions within the synthesized compounds were confirmed by the FTIR. Figure 5 presents the FTIR spectra of three samples: chitosan matrix (black spectrum), Cu–gallate (blue spectrum), and the Cu–gallate/CS/IL membrane (red spectrum). The FTIR spectrum of pure chitosan presents two main relatively broad bands at 3443 cm^–1^ due to hydroxyl (–OH) and 1639 cm^–1^ because of carbonyl (C=O) groups. The Cu–gallate spectrum shows that a significantly enhanced 3426 cm^–1^ broadband is attributed to the additional –OH group contribution from the gallic acid linker, whereas the chelation between the copper metal and the carboxyl (–COOH) group has resulted in a peak at 1640 cm^–1^. Two more bands were observed at 1544 (asymmetric) and 1395 cm^–1^ (symmetric) attributed to the C–O stretching from the carboxylate (OCO–) group, which matches the free gallic acid linker (1540 and 1440 cm^–1^) from reported data.^55^ These results indicate that both the hydroxyl and carboxylate groups of GA were involved in coordination with the copper ion.

**Figure 5:**
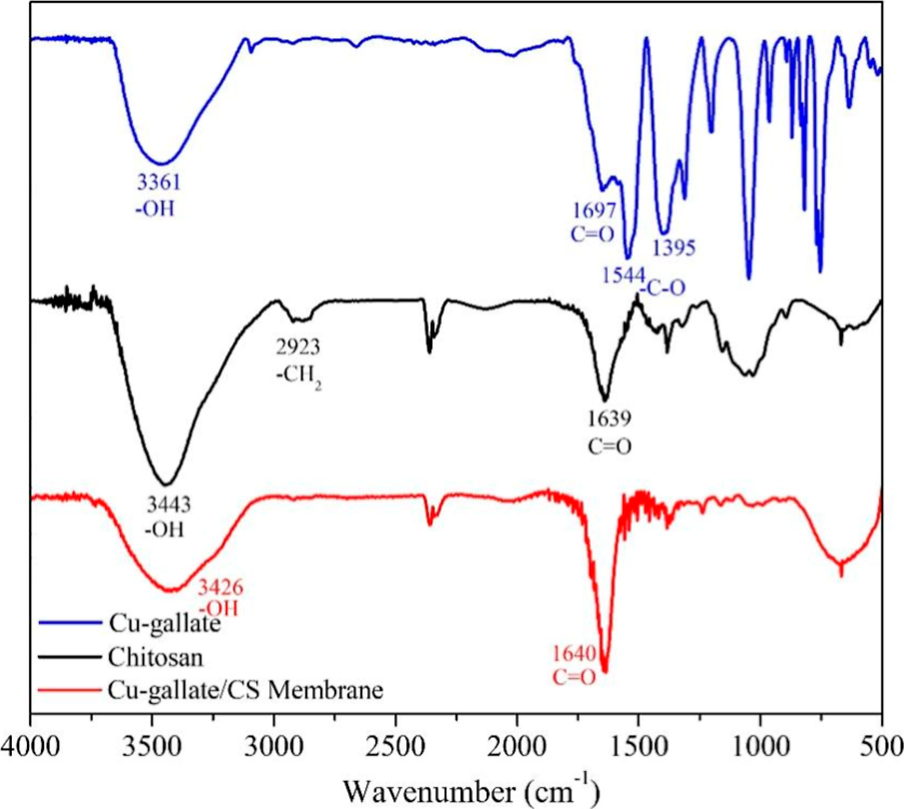
FT-IR spectra of chitosan, Cu–gallate pure MOF, and Cu–gallate/CS membrane.

For the Cu–gallate/CS/IL membrane spectrum, the 3361 cm^–1^ broad band arises from –OH groups from both the gallate linker and the chitosan. The C=O group within the membrane is confirmed by a band at 1697 cm^–1^, indicating the Cu–gallate MOF is stable within the membrane.

### 3.1 Gas Sensing Performance

The acetone sensor device was constructed using the approach previously developed by our group.^12,13,37^ To prepare sensing membranes, different concentrations of Cu–gallate MOF (1%, and 3%) were incorporated into a matrix of chitosan/ionic liquid (CS/IL). The response of these membranes was then evaluated at 100 ppm across various gases, including acetone. The response of the Cu–gallate/CS/IL membrane toward acetone gas was tested at varied temperatures (25–80 °C) and with varying voltage biases (1–5 V). The membrane with 3 wt % Cu–gallate exhibited the highest response toward acetone at 80 °C and 5 V bias voltage. Equation 1 was used to calculate the sensor response (S) as follows:

1 $$S\left(\%\right)=\frac{R_{g}-R_{a}}{R_{a}}\times 100=\frac{\Delta R}{R_{a}}\times 100$$

where *R*_a_ and *R*_g_ denote air sensor resistance and the resistance under test gas exposure, respectively. To assess response, the prototype was subjected to varying acetone gas concentrations under optimum conditions (5 V and 80 °C) (Figure 6A), which showed a high response toward acetone gas. The sensor successfully detected acetone with the detection limit of 0.25 ppm (Figure 6B). The performance of the Cu–gallate/CS/IL membrane toward different acetone concentrations has also been examined and is presented in Figure S3.

**Figure 6:**
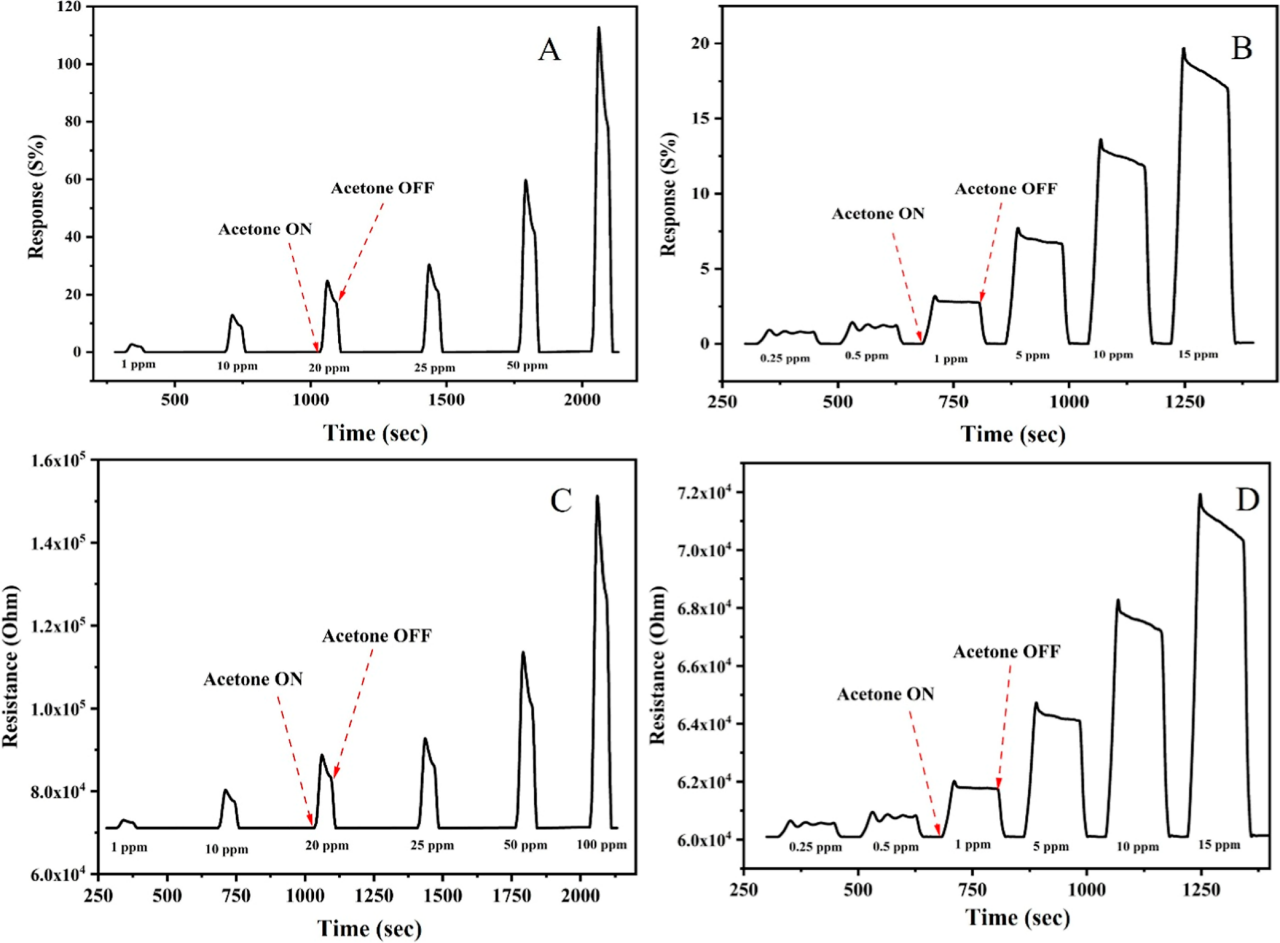
Gas sensing performance of the 3 wt % Cu–gallate/CS/IL membrane at 80 °C toward acetone gas. (A,B) Sensor response at acetone concentrations ranging from (A) 1 to 100 ppm and (B) 0.25 to 15 ppm. (C,D) Response transients corresponding to acetone concentrations of (C) 1–100 ppm and (D) 0.25–15 ppm.

To evaluate the sensing performance of different doping percentages of Cu–gallate in the chitosan matrix, tests were conducted at two different doping percentages 1% and 3% Cu–gallate MOF under the same conditions, with 100 ppm acetone, 5 V, and a temperature of 80 °C represented in Table 1. The sensor exhibited a higher response of 96.73 ± 0.89% for 3% in comparison to the lower response of 12.40 ± 1.43% for 1% Cu–gallate MOF concentration. To make a more detailed comparison, an additional bare sensor that comprises a mixture of CS/IL alone without Cu–gallate MOF was constructed. As illustrated in Figure S4, the sensor displayed an 18.83% response to 100 ppm of acetone, while the 3% Cu–gallate/CS/IL sensor exhibited a much higher 120% response. This highlights the substantial improvement in sensor performance due to the inclusion of the MOF.

**Table 1:** Comparison of Sensing Performance at Different Doping Percentages for 100 ppm Acetone Gas at 80 °C

doping wt % of Cu-gallate in CS/IL matrix	sensing response S %
1%	12.40 ± 1.43
3%	96.73 ± 0.89

To determine the optimal operating temperature, an extra measurement was performed. As shown in Figure 7, the sensor response (S %) was analyzed against the temperature and acetone concentration with each data point representing the average response at a specific temperature and gas concentration. The results indicate a good response to acetone gas, even at 25 °C. The error bars show a low standard deviation, remaining below 5% for each point, demonstrating the sensor’s reliability. At lower temperatures (25, 40, and 60 °C), however, the response to acetone was noticeably reduced, as shown in Figures S5 and S6. Raising the temperature to 80 °C yielded the highest average response at approximately 110% at an acetone concentration of 100 ppm, outperforming responses observed at lower temperatures. These findings emphasize the significant influence of temperature on sensor performance. At elevated temperatures, both acetone gas molecules and the sensor’s active sites gain additional thermal energy, enhancing their mobility. This increase in energy facilitates greater molecular diffusivity, promoting more frequent and intense interactions between the acetone molecules and the sensor surface. As a result, adsorption and reaction processes are accelerated, leading to a markedly improved sensor response. This operating temperature, while above physiological levels, is compatible with diagnostic devices that incorporate preconditioned sampling chambers or localized heating to enhance sensor performance.

**Figure 7:**
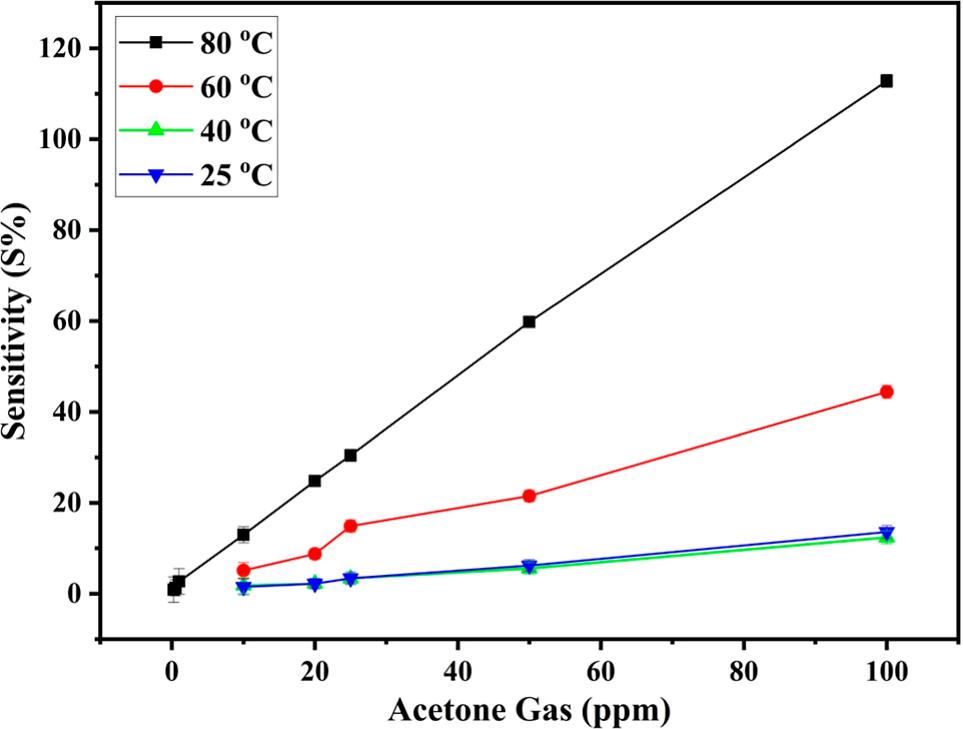
Sensor’s response at different temperatures for different ppm of acetone gas.

Selectivity is an important parameter in evaluating the sensor performance. To assess this, the sensor’s response to other gases (100 ppm) at 80 °C was tested to determine its ability to selectively detect acetone. The selectivity test, shown in Figure 8, was conducted with five available gases: H_2_S, C_2_H_4_, H_2_, CO, and CO_2_. Compared with the other gases evaluated, the sensor showed the most pronounced response to acetone, with the remaining gases yielding responses under 40%.

**Figure 8:**
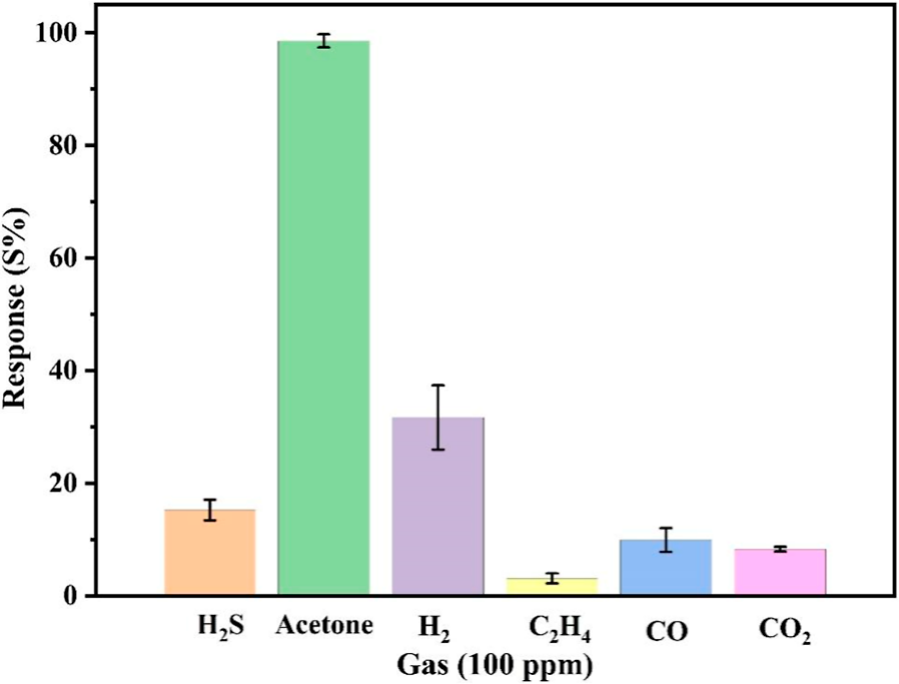
Selectivity of the Cu–gallate/CS/IL membrane with comparison to other gases at 80 °C.

Repeatability and stability are other crucial parameters for sensors. To evaluate this, the sensor was subjected to 100 ppm of acetone over five cycles, with internment synthetic air flushing to remove left over acetone. The sensor demonstrated a repeatability of 98.55 ± 1.16% (Figure 9A). On the other hand, these tests were repeated for 21 days continuously for investigating stability of the probe. Irrespective of the number of days, the sensor exhibited a stable response of 96.73 ± 0.89%, as shown in Figure 9B. The measured responses remained very close to the initial values recorded at the same temperature and acetone concentration. These findings validate the outstanding reproducibility and long durability of the Cu–gallate/CS/IL sensor. Furthermore, PXRD (Figure S7) clearly demonstrated that the Cu–gallate/CS/IL membrane retained crystallinity, even after the repeated measurements.

**Figure 9:**
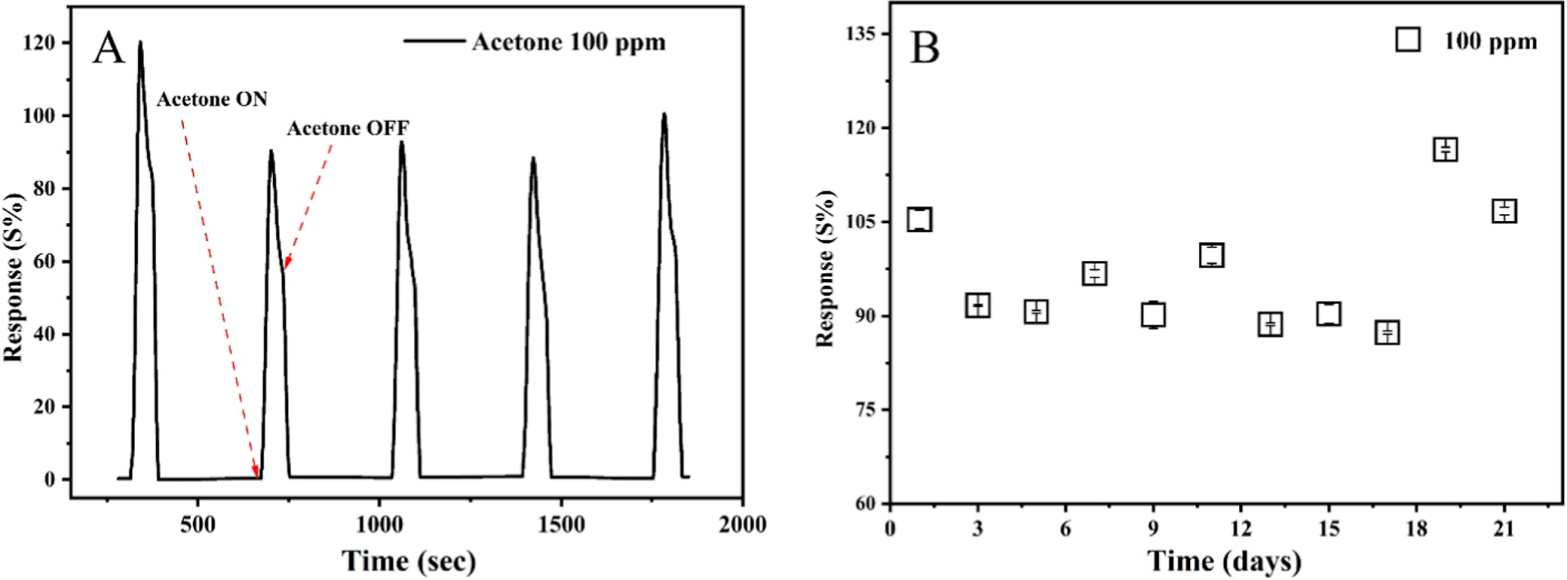
(A) Repeatability and (B) long-term stability of the Cu–gallate/CS/IL composite membrane at 80 °C toward 100 ppm of acetone gas.

In addition, the response and recovery times were also measured. The response time is the time taken by the sensor to reach a maximum response of 90% after exposure to the target gas, while the recovery time is the time taken by the sensor to return from 90 to 10% after ceasing the target gas. As illustrated in Figure 10, the sensor took 27 ± 1.96 s to reach maximum response with a recovery time of 10.50 ± 0.98 s.

**Figure 10:**
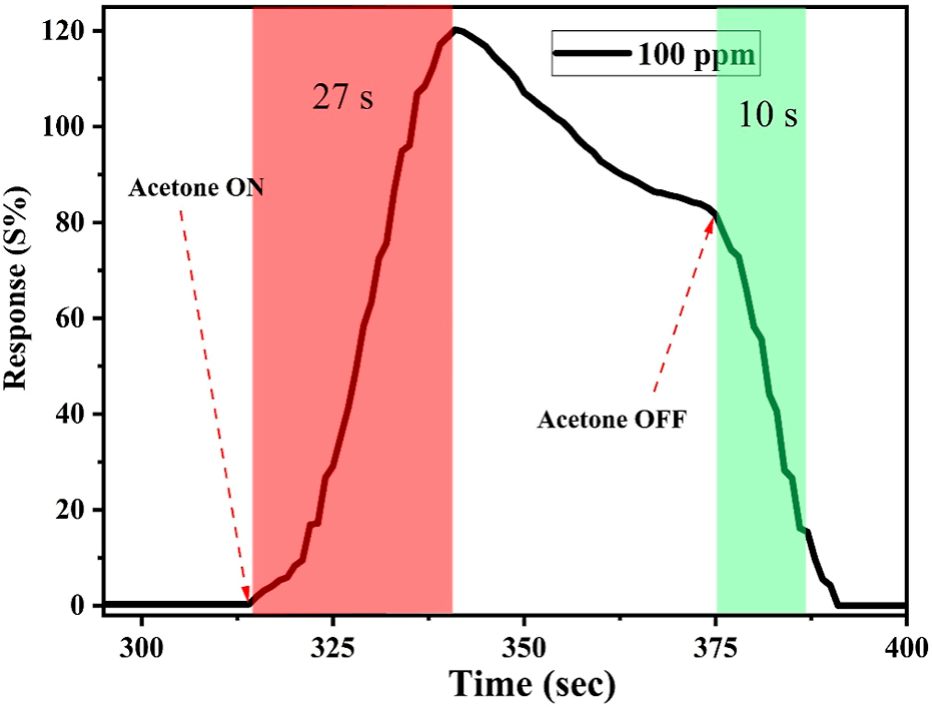
Response and recovery time of the prepared Cu–gallate/CS/IL membrane at 80 °C toward 100 ppm of acetone gas.

To investigate how the relative humidity (RH) influences gas sensor behavior, tests were conducted at various humidity levels (0–90%). A commercial humidity meter equipped with a probe was used to monitor the RH inside the gas chamber during the sensing tests. The Cu–gallate/CS/IL membrane sensor was tested under these RH conditions at 80 °C in the presence of 100 ppm of acetone. The sensor response, as illustrated in Figure 11, decreases steadily as a function of humidity. Specifically, the response decreased from 85% to 54% (at 50% relative humidity), and at 90% relative humidity, it dropped further to 13%. For context, Slawek et al.^57^ reported that the humidity levels present in the exhaled breath in case of both healthy and asthmatic individuals typically fall between 50% and 74%. Within this range, the Cu–gallate sensor is expected to maintain a response between 54% and 37%, demonstrating consistent and reliable performance even under high-humidity conditions.

**Figure 11:**
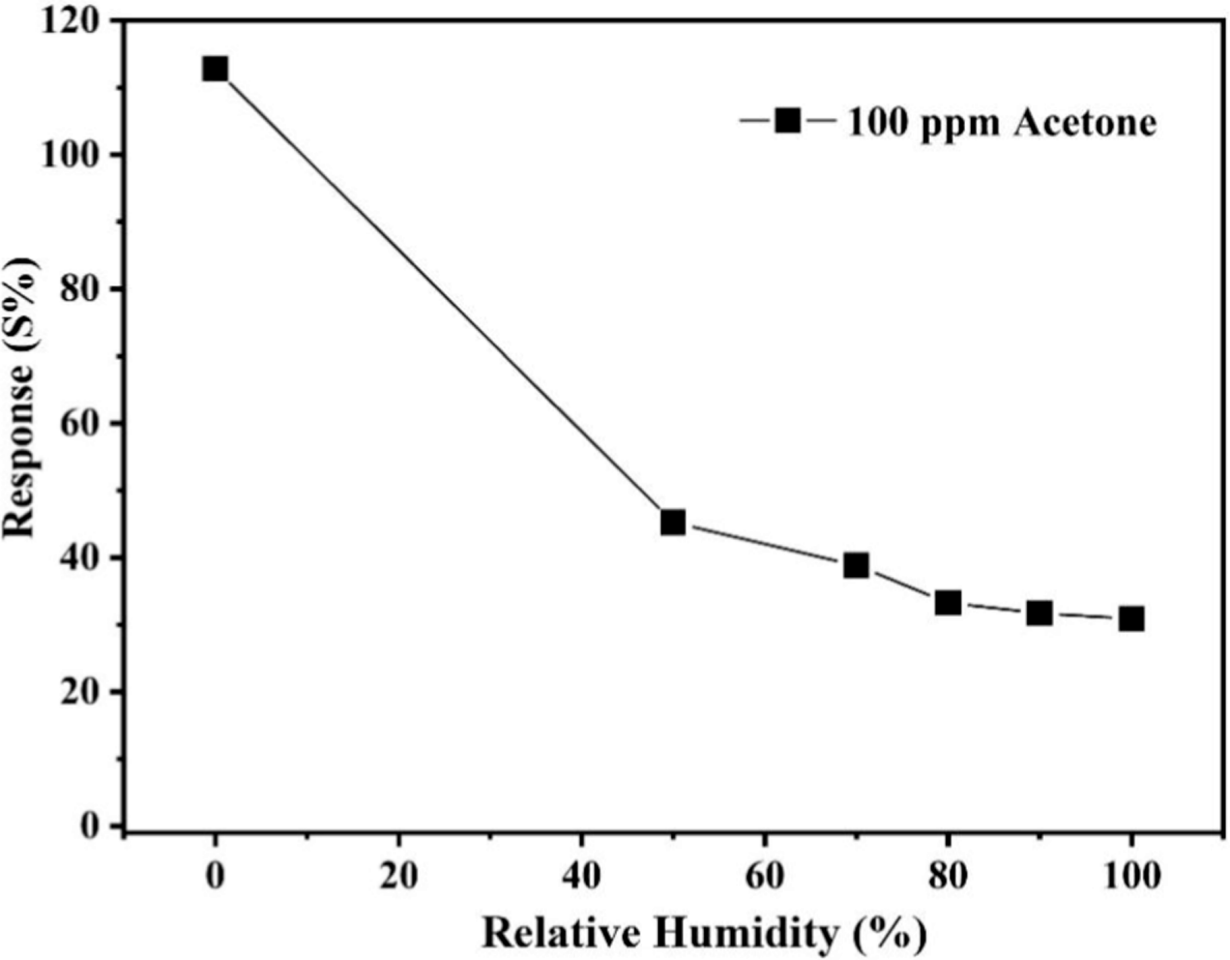
Impact of relative humidity on the sensing performance of the Cu–gallate/CS/IL membrane at 80 °C toward 100 ppm of acetone gas.

Recent MOF-based gas sensors have focused on high sensitivity at low temperatures to enhance their practical applicability. A Cu–MOF/PVA/IL membrane detected H_2_S at room temperature with a 1 ppm limit and 12 s response [24]. UiO-66-NH_2_ and Bigallate integrated into CS/IL matrices achieved acetone detection at 60 °C with limits of 1 and 10 ppm, respectively, and response/recovery times of 23/18 s and 15/3 s, respectively.^12,13^ While effective, their detection limits remain in the ppm range.

The Cu–gallate/CS/IL membrane developed here advances the field by achieving an ultralow acetone detection limit of 0.25 ppm at 80 °C, with a rapid response/recovery of 27/10 s and robust stability under medium humidity. This performance highlights the synergistic effect of Cu–gallate MOFs with chitosan and ionic liquids, delivering a highly sensitive, fast, and reliable sensing platform. Compared with previous reports (Table 2), this study sets a new benchmark for subppm acetone detection, offering a versatile platform for noninvasive medical diagnostics and environmental monitoring.

**Table 2:** Summary of the Performance of Reported Sensors from the Literature Compared to Our Work

material	temp (°C)	conc (ppm)	response/recovery time (s)	reference
ZIF-67	250	50	NR	^58^
ZIF-67/ZIF-8	275	1	NR	^58^
Bi(HHTP)	25	41.2	NR	^59^
Bigallate membrane	60	10	15/3	^13^
UiO-66-NH_2_/CS membrane	60	1	23/18	^12^
Cu–gallate/CS/IL membrane	80	0.25	27/10	this work

NR—Not Reported.

In Figure 12, we propose a mechanism for acetone sensing via the Cu–gallate/CS/IL membrane. Abu-Hani et al.^8^ investigated the performance of the CS/IL membrane without MOF inclusion. In our system, hydrogen bonds form between the chitosan matrix and the ionic liquid (IL), which plays a key role in improving ionic conductivity and facilitating charge transport, thereby amplifying the sensor’s electrical response to acetone.^8^ Additionally, the chemical structure of the Cu–gallate MOF, rich in oxygen and nitrogen atoms, offers further sites for hydrogen bonding with chitosan. Upon exposure to acetone gas, the polar acetone molecules interact with this hydrogen-bonded network, enhancing the proton conductivity across the Cu–gallate MOF/CS/IL membrane matrix.

**Figure 12:**
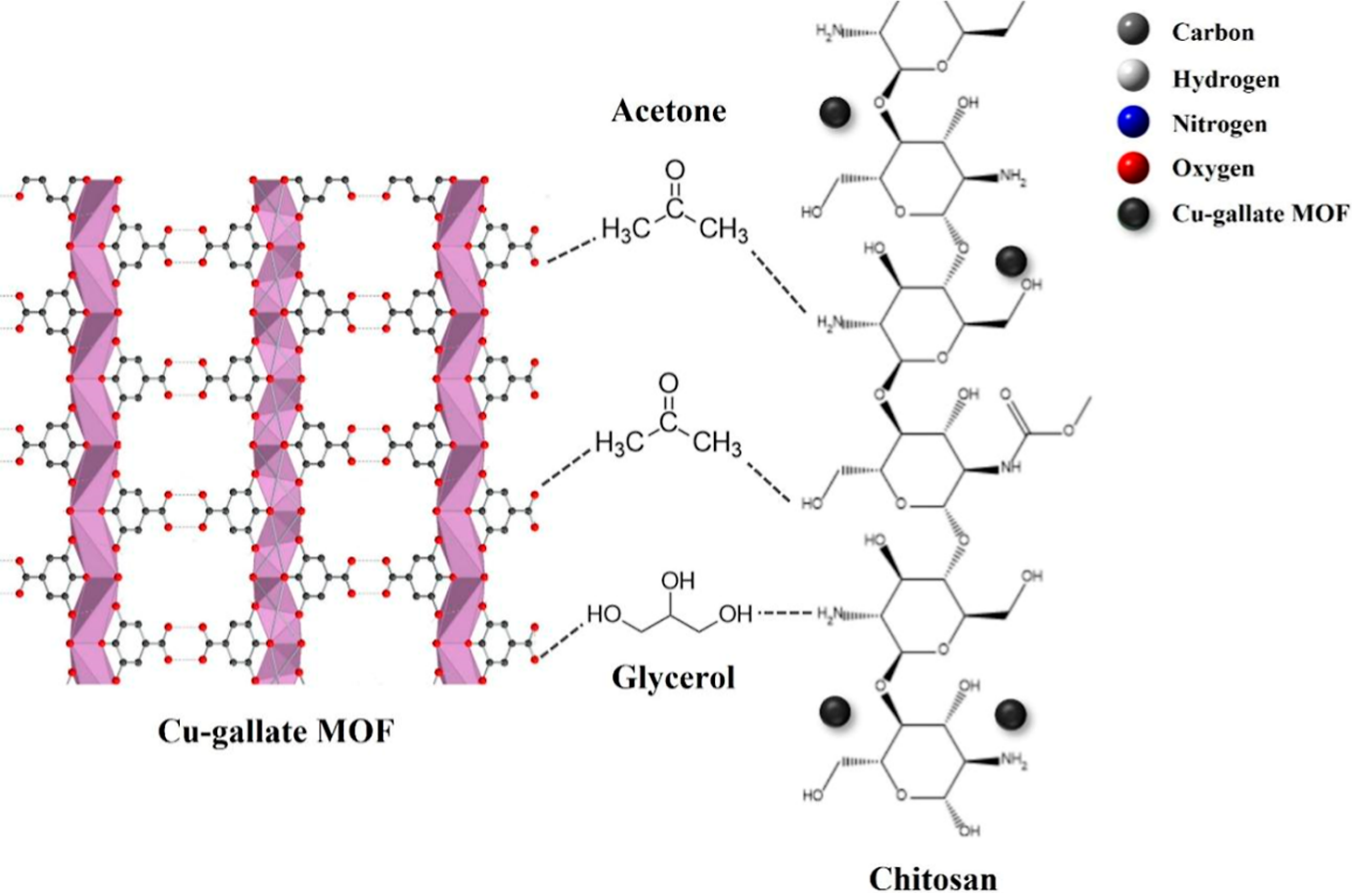
Proposed sensing mechanism of the Cu–gallate MOF/CS/IL membrane with acetone gas.

## 4 Conclusion

In conclusion, this study presents the successful development of a Cu–gallate MOF/chitosan/ionic liquid composite membrane for selective acetone detection in human breath. The sensor exhibits a low detection limit of 0.25 ppm at 80 °C, along with rapid response and recovery times, excellent selectivity, and stable long-term performance. Incorporation of chitosan improved the membrane’s conductivity and enhanced acetone selectivity through hydrogen-bonding interactions, underscoring the synergistic role of the polymer–MOF combination. Furthermore, the sensor maintained a reliable functionality under moderate humidity conditions. Overall, these findings highlight Cu–gallate MOF-based mixed-matrix membranes as a promising and cost-effective platform for noninvasive medical diagnostics and advanced gas-sensing technologies.

## Supplementary Material



## Notes

Energy-dispersive X-ray analysis and differential thermogravimetric analysis of the Cu–gallate/CS membrane and additional figure illustrating the membrane’s response to acetone gas (PDF)
