# Supplementary material for: Cu–Gallate MOF–Chitosan Hybrid Membrane for Low-Power, Non-Invasive Acetone Sensing: Toward Early-Stage Diabetes Detection
Source: ACS Omega. 2025 Nov 24;10(48):59885–94. doi: 10.1021/acsomega.5c09912 (PMC12772395; doi:10.1021/acsomega.5c09912)
Supplement: Supplementary file 1 [file ao5c09912_si_001.pdf]

SUPPORTING INFORMATION

Cu–Gallate MOF–Chitosan Hybrid Membrane for Low-Power, Non-Invasive Acetone Sensing: Toward Early-Stage Diabetes Detection

Lamia A. Siddig,[a,b], Yaser E. Greish[b] , Ashraf Ali[c], Khadega A. Al-Maqdi[b], Abdul Hakeem Deshmukh[a], Naser N. Qamhieh[a], and Saleh T. Mahmoud[a,]*

[a] Department of Physics, United Arab Emirates University, Al-Ain 15551, United Arab Emirates.

[b] Department of Chemistry, United Arab Emirates University, Al-Ain 15551, United Arab Emirates

[c] Department of Physics, Khalifa University of Science and Technology, Abu Dhabi, P. O. Box 127788, United Arab Emirates.

* Corresponding Author: E-mail address: saleh.thaker@uaeu.ac.ae (S.T.M).

TABLE OF CONTENTS

Section I: Energy-Dispersive X-ray (EDX)…...……………………………………..…S3

EDX spectrum of Cu-gallate MOF and Cu-gallate/CS membrane.…..……….................S3

EDX table of weight and atomic percentages ………….………………..……................S3

Section II: Differential thermogravimetric analysis (DTG)……….………...…..……S4

Section III: Response of the Cu-gallate/CS membrane ……………..…….…...…..…S5

Response of the Cu-gallate/CS membrane to different acetone concentrations (A) from 1 ppm to100 ppm and (B) from 0.25 ppm to 15 ppm………….....................S5

The response of the chitosan/IL membrane and the Cu-gallate /CS membrane to 100 ppm acetone at 60 °C...S5

Summary of the response of Cu-gallate/CS membrane at 25 °C, 40 °C, 60 °C, and 80 °C…………………………………………………….……………………......S6

Response of the Cu-gallate/CS membrane at 25°C, 40°C, 60°C, and 80°C…...……........S6

PXRD spectrum for the Cu-gallate/CS membrane after the repeatability test…………...S7

Section I: Energy-dispersive X-ray Spectroscopy (EDX)

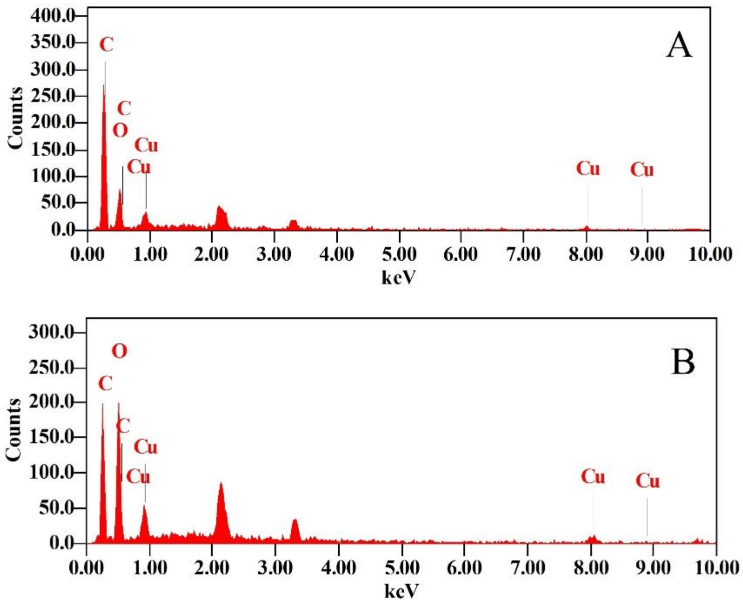

Figure S1. EDX data of (A) Cu-gallate MOF and (B) Cu-gallate/CS membrane.

Table S1. Elemental analysis of as-prepared Cu-gallate MOF and Cu-gallate/CS composite membrane as depicted from its SEM micrograph

Element	C	N	O	Cu
Mass% of Cu-gallate MOF	34.42	-	24.65	40.93
Atom % Cu-gallate MOF	56.74	-	30.51	12.76
Mass% of Cu-gallate/CS composite	39.66	4.71	35.91	19.72
Atom % of Cu-gallate/CS composite	53.31	5.43	36.25	5.01

Section II: Differential thermogravimetric analysis (DTG)

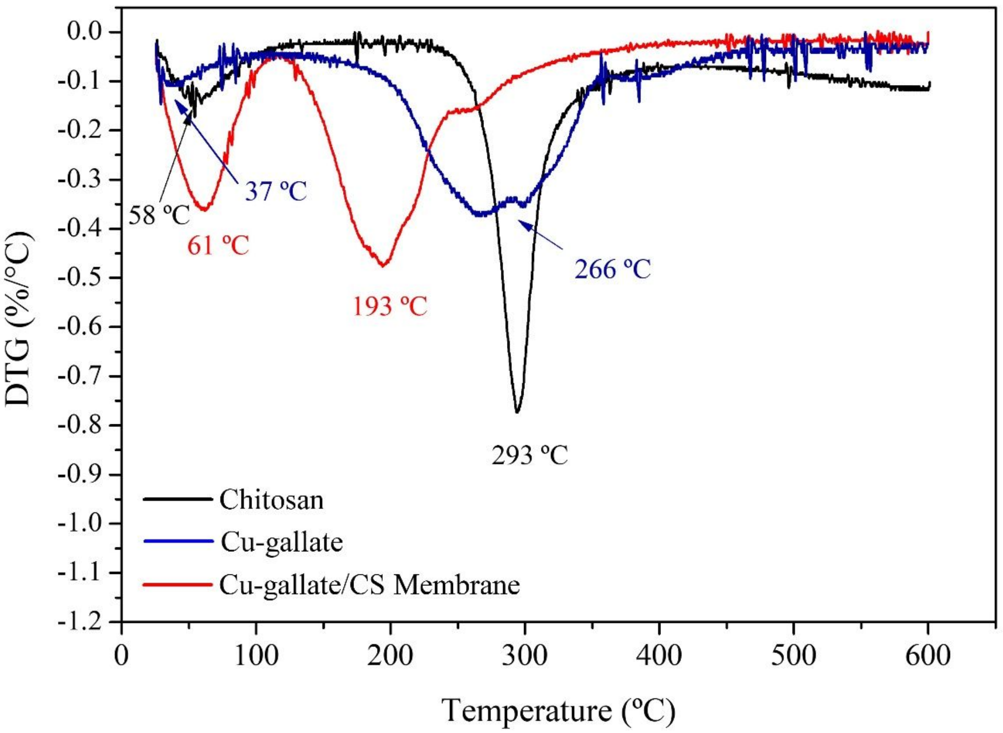

Figure S2. DTG thermograms of pure chitosan, Cu-gallate MOF and Cu-gallate /CS composite membrane.

Section III: Response of the Cu-gallate/CS membrane

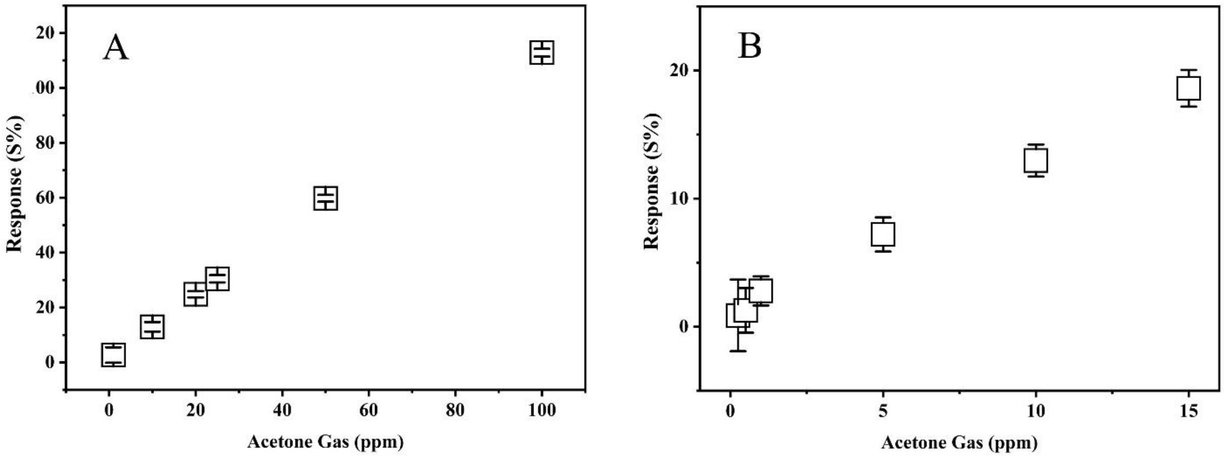

Figure S3. Response of the Cu-gallate/CS membrane to different acetone concentrations (A) from 1 ppm to100 ppm and (B) from 0.25 ppm to 15 ppm.

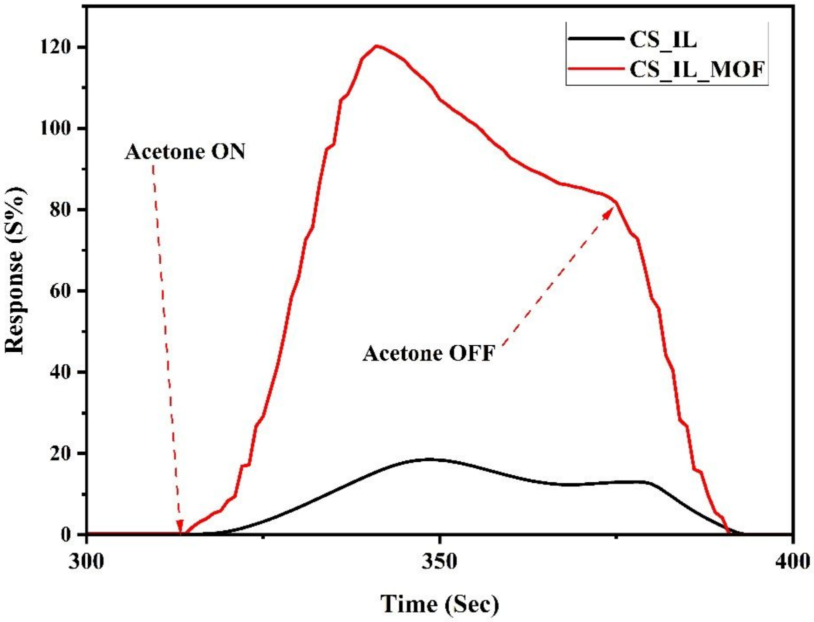

Figure S4. The response of the chitosan/IL membrane and the 3% Cu-gallate/CS membrane to 100 ppm acetone at 80 °C.

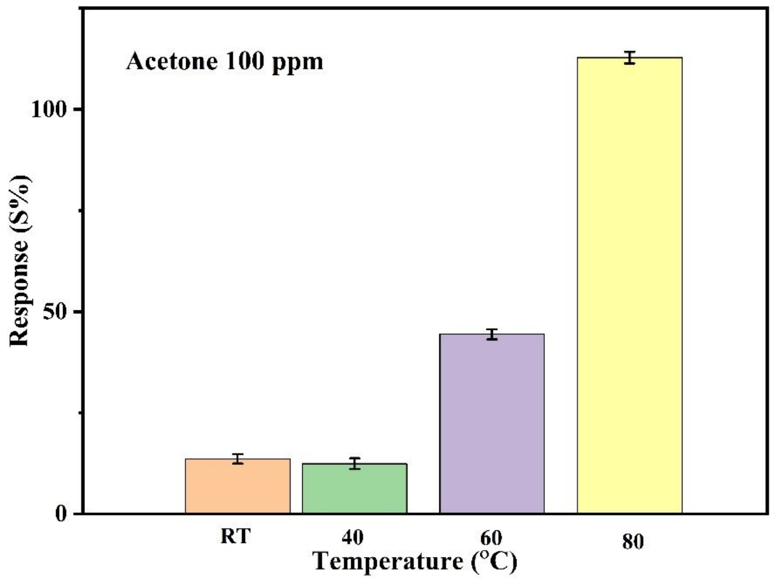

Figure S5. Summary of the response of Cu-gallate/CS membrane at 25 °C, 40 °C, 60 °C, and 80 °C.

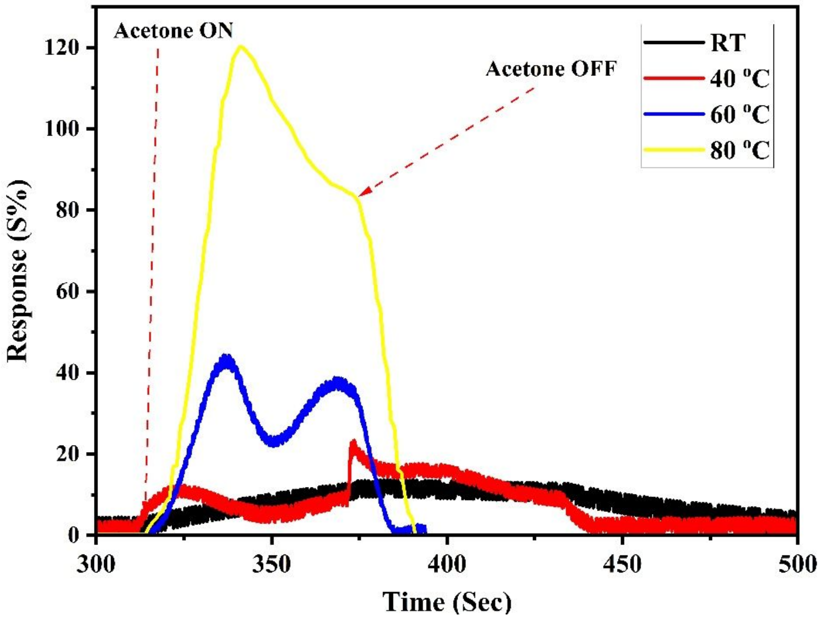

Figure S6. Response of the Cu-gallate/CS membrane at 25 °C, 40 °C, 60 °C, and 80 °C.

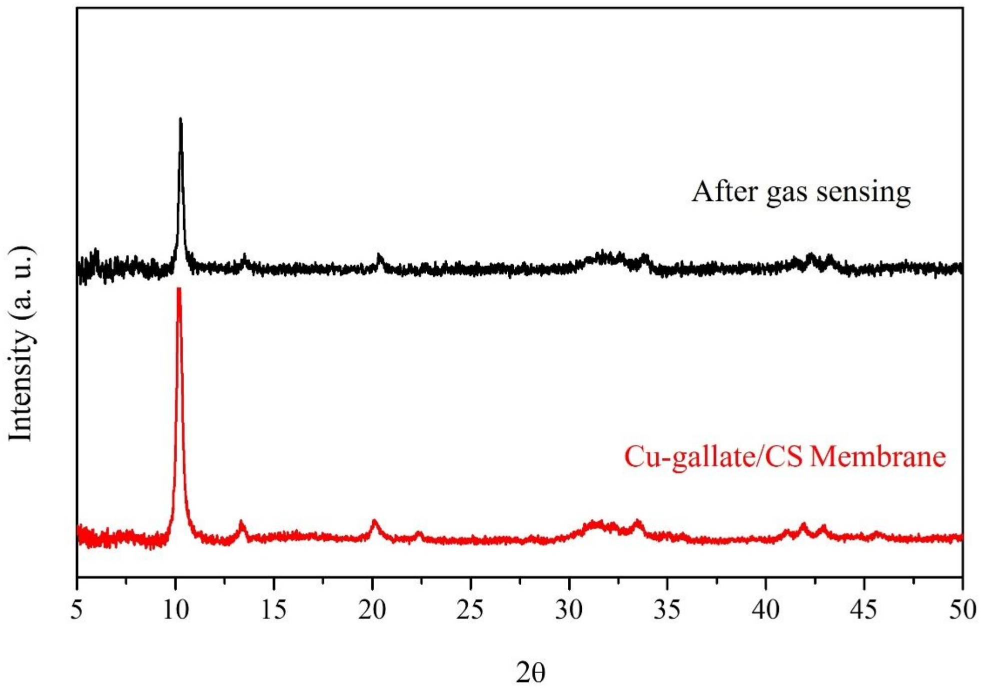

Figure S7. PXRD patterns of the Cu-gallate/CS membrane before and after the repeatability test.
